# Transcriptome Profiling of Developing Ovine Fat Tail Tissue Reveals an Important Role for *MTFP1* in Regulation of Adipogenesis

**DOI:** 10.3389/fcell.2022.839731

**Published:** 2022-03-08

**Authors:** Jiangang Han, Sijia Ma, Benmeng Liang, Tianyou Bai, Yuhetian Zhao, Yuehui Ma, David E. MacHugh, Lina Ma, Lin Jiang

**Affiliations:** ^1^ Laboratory of Animal Genetics, Breeding and Reproduction, Ministry of Agriculture, Institute of Animal Sciences, Chinese Academy of Agricultural Sciences (CAAS), Beijing, China; ^2^ Animal Genomics Laboratory, UCD School of Agriculture and Food Science, UCD College of Health and Agricultural Sciences, University College Dublin, Dublin, Ireland; ^3^ Agricultural College, Ningxia University, Yinchuan, China; ^4^ National Germplasm Center of Domestic Animal Resources, Ministry of Technology, Institute of Animal Sciences, Chinese Academy of Agricultural Sciences (CAAS), Beijing, China; ^5^ UCD Conway Institute of Biomolecular and Biomedical Research, University College Dublin, Dublin, Ireland; ^6^ Institute of Animal Science, Ningxia Academy of Agriculture and Forestry Sciences, Yinchuan, China

**Keywords:** MTFP1, fat tail development, adipogenesis, RNA-seq, mitochondrion

## Abstract

Fat-tail sheep exhibit a unique trait whereby substantial adipose tissue accumulates in the tail, a phenotype that is advantageous in many agroecological environments. In this study, we conducted histological assays, transcriptome analysis and functional assays to examine morphogenesis, characterize gene expression, and elucidate mechanisms that regulate fat tail development. We obtained the microstructure of tail before and after fat deposition, and demonstrated that measurable fat deposition occurred by the 80-day embryo (E80) stage, earlier than other tissues. Transcriptome profiling revealed 1,058 differentially expressed genes (DEGs) with six markedly different expression trends. GSEA enrichment and other downstream analyses showed important roles for genes and pathways involving in metabolism and that mitochondrial components were specifically overexpressed in the fat tail tissue of the 70-day embryo (E70). One hundred and eighty-three genes were further identified by leading edge gene analysis, among which, 17 genes have been reported in previous studies, including *EEF1D*, *MTFP1*, *PPP1CA*, *PDGFD*. Notably, the *MTFP1* gene was highly correlated with the expression of other genes and with the highest enrichment score and gene expression change. Knockdown of *MTFP1* in isolated adipose derived stem cells (ADSCs) inhibited cell proliferation and migration ability, besides, promoted the process of adipogenesis *in vitro*.

## Introduction

Based on tail morphology, domestic sheep (*Ovis aries*) can be assigned to three biological categories: fat-tail sheep, thin-sheep and fat-rump sheep (Kalds et al., 2021). Among mammals, only sheep deposit extensive fat tissue during development of the tail (Al-Rehaimi et al., 1989). With regard to humans, excessive fat deposition and obesity inevitably leads to negative health effects, including cardiovascular disease, hypertension, diabetes, increased predisposition to certain types of cancer (Gonzalez-Muniesa et al., 2017). Certain breeds of domestic sheep, however, have evolved a unique fat-tail phenotypic trait because of natural selection and human-mediated breeding to enhance adaptive mechanisms for inhibiting fat metabolism and stimulating localized fat storage as an energy source during food scarcity and for human consumption. During recent millennia, as a consequence of this advantageous trait, fat-tailed sheep breeds have expanded, diversified and spread across Eurasia and now represent a substantial proportion of the global sheep population (Rocha et al., 2011).

Currently, modern intensive and semi-intensive production systems have precluded the need for sheep to deposit significant fat in the tail tissue to survive and be productive in harsh environments (Bakhtiarizadeh and Salami, 2019). Furthermore, the fat-tail trait is not as desirable for sheep breeders because of high feed-meat ratios combined with low prices, reproductive problems caused by large tails, reduced carcass quality (Yousefi et al., 2012), and the relative unpopularity of fatty meat in modern human diets. Consequently, all these factors have incentivized breeding and production of sheep with thinner and smaller tails and have prompted researchers to explore the physiological and molecular mechanisms underpinning tail fat deposition.

Among various population genetics and transcriptome studies targeting the fat-tail trait, several genes (for example, *PDGFD*, *BMP2* and *TBXT*) have been identified as promising candidate genes (Kalds et al., 2021) and almost all of these studies were conducted in adult sheep. Based on resequencing data or SNP data from various sheep breeds worldwide with different tail types, the *PDGFD* gene has been identified as the most important candidate gene associated with tail morphology (Wei et al., 2015; Pan et al., 2019; Dong et al., 2020; Li et al., 2020); however, the strongest signals identified within *PDGFD* gene are different. In addition, several transcriptomics studies have focused on mRNA, lncRNA, and microRNA expression patterns in ovine tail tissue (Wang et al., 2014; Li et al., 2018; Ma et al., 2018; Bakhtiarizadeh et al., 2019; Wang et al., 2021; Zhang et al., 2021). For example, the most recent study (Zhang et al., 2021) compared the mRNA profile between fat-rump and thin-tail sheep and identified 198 DEGs that exhibited increased expression, which enriched for adipocytokine and PPAR signaling pathways.

A substantial body of research work has been published on sheep fat-tail biology and development (Kalds et al., 2021; Pourlis, 2011); however, to-date the genetic architecture of this complex trait has not been investigated. In addition, it is important to keep in mind that prior to birth, substantial fat already exists in tail tissue and that the regulatory mechanisms governing mammalian tail development remain poorly understood. Consequently, in the present study we address these knowledge gaps through investigation of the early stages of fat-tail development.

The gestation period for sheep is normally 140–150 days, depending on breed; therefore, we inferred that the mid-period of gestation (∼70 d) would encompass important developmental changes in fat tail tissue. To clarify and better understand the molecular mechanisms underpinning tail morphogenesis, we selected embryonic tail tissue of fat-tailed sheep at three different stages that encompass tail development (60-day, 70-day and 80-day embryo: E60, E70 and E80, respectively). In addition, as controls, we also examined adult fat-tail tissue (Fat) and embryonic tail tissue from thin-tailed sheep (Suffolk) at E70 (E70_SFK).

We conducted morphological and comparative transcriptomics analyses using these samples. Our results allowed us to identify a key time point in fat-tail development, tail development patterns, and a series of differentially expressed genes (DEGs) involved in energy metabolism. Furthermore, we integrated our results with previously reported genes and performed functional verification for the most significantly differently expressed gene (*MTFP1*) in isolated adipose derived stem cells (ADSCs).

## Materials and Methods

### Animal Procedures and Sample Collection

All animal experimental procedures were inspected and reviewed by the Animal Welfare and Ethic Committee of the Institute of Animal Science, Chinese Academy of Agriculture Sciences (approval number: IAS 2021-73) and conduced according to the guidelines established by the Institutional Animal Care and Use Committee of the Chinese Academy of Agriculture Sciences. Animal and sample collection work was performed at the National Tan Sheep Conservation Farm. A total of 15 female Tan sheep (fat-tail sheep) and five female Suffolk sheep (thin-tail sheep) aged from three to 4 years old were maintained under the same feeding regimen, management and environment.

All sheep were treated with estrus synchronization and artificial insemination. In brief, this involved the following: 1) a controlled internal drug releasing (CIDR) progesterone implant for 11 days; 2) removal of the progesterone implant and injection of 1 ml cloprostenol (0.1 mg/ml); 3) estrus identification after 48 h; 4) only sheep that were in estrus at the same time were used for artificial insemination; and 5) A B-ultrasonic machine (Gandaofo, Henan, China) was used to evaluate pregnancy status after approximately 30 days. When the Tan sheep embryos developed to the 60-day (E60), 70-day (E70) and 80-day (E80) stages individually, and the Suffolk sheep embryo reached the 70-day (E70_SFK) stage, the pregnant ewes were transferred to the slaughterhouse. Samples of embryonic tail, skin, heart, muscle, liver, and kidney were collected from at least three individual animals, and fat tissues from the tail of every adult sheep were also harvested.

### Histological Analysis

Samples were immersed in 4% paraformaldehyde solution for fixation, dehydrated in a graded ethanol series, cleaned with xylene and embedded in paraffin wax. Using a RM2255 Automated Rotary Microtome (Leica, Wetzlar, Germany), the paraffin-embedded tissue blocks were sectioned into 5 μm-thick slices, which were then stained using hematoxylin and eosin (HE staining). For oil red staining, tissue was frozen with embedding medium swiftly after fixation and cut into sections as described above; it was then stained with oil red O solution. A BX51 microscope (Olympus, Shinjuku, Japan) and a DP72 digital imaging system (Olympus, Shinjuku, Japan) were used to visualize stained sections.

### RNA Isolation and Sequencing

The tail tissues of Tan sheep at four time points (E60, *n* = 4; E70, *n* = 3; E80, *n* = 4; Adult, *n* = 3) and the tail tissues of Suffolk sheep at E70 (E70_SFK, *n* = 4) were used for RNA extraction with the RNeasy lipid tissue Kit (Qiagen, Hilden, Germany). The concentration and quality of RNA samples were evaluated using an Agilent 2,100 Bioanalyzer (Agilent, California, United States). Samples with RIN values higher than 7.0 and concentrations greater than 40 ng/ul were used for RNA-seq. The mRNA selection, library preparation and sequencing were performed by the BerryGenomics Company (Beijing, China) and sequencing was performed on the HiSeq X Ten high-throughput sequencing (HTS) platform (Illumina, California, United States).

### Quality Control and Genome Mapping for mRNA Reads

The NGS QC Toolkit (v2.3.3) (Patel and Jain, 2012) was used to perform quality control of the raw data, including removal of reads with overall quality scores less than 20 according to the Phred+33 scale for at least 70% of the bases and adapter sequences. The remaining clean reads were mapped to the sheep reference genome (Oar v4.0) (Jiang et al., 2014) using the TopHat2 software tool (v2.1.1) (Kim et al., 2013) with default parameters.

### Assembly of Transcripts and Differential Expression Analysis

Assembly of the mapped reads and transcriptome quantification (in Fragments Per kilobase of transcript per Million mapped reads; FPKM) were performed using the Cufflinks package (v2.2.1) (Trapnell et al., 2012) with the sheep reference genome and annotation file. The Cuffmerge program was then used to generate a new genome annotation file with unified annotation information. Following this, the Cuffdiff program was used to identify DEGs between experimental groups. We removed genes that, at least in one group, exhibited mean FPKM value less than 0.5. Genes that exhibited absolute log_2_ fold change values ≥ 1 and FDR-adjusted *q* values <0.05 were considered to be differentially expressed.

### Principal Component Analysis, Venn, and Heatmap Analyses

The gmodels package was used to perform principal component analysis (PCA) in R (version 3.6.1) with the FPKM values for all annotated transcripts from the eighteen transcriptomes. The heatmap and Venn diagrams were generated using the FPKM values and gene symbols of corresponding DEGs with the Pheatmap and VennDiagram packages in R (version 3.6.1).

### Hierarchical Clustering Analysis

The standardized and centralized FPKM values were used for hierarchical analysis, with cluster numbers determined by the Calinsky criterion. Using the “timeclust” parameter of the TCseq package (Gu, 2021) to perform the hierarchical analysis with corrected FPKM. The cluster dendrogram was divided using the “complete” parameter to classify the genes based on expression trend.

### Pathway Enrichment Analysis

Gene ontology (GO) and Kyoto Encyclopedia of Genes and Genomes (KEGG) enrichment analyses was implemented using the clusterProfiler package (Wu et al., 2021). The GSEA software (version 4.0.2) (Subramanian et al., 2005), which uses predefined gene sets from the Molecular Signatures Database, was applied to perform gene set enrichment analysis based on FPKM values for the E70 and E70_SFK experimental groups. In this study, we used H: hallmark gene sets, CP:KEGG: KEGG gene sets and C5: GO gene sets as molecular signatures databases and gene sets with FDR *q* values <0.05 were considered to represent significant pathways. The minimum and maximum criteria for selection of gene sets from the collection were 10 and 500 genes, respectively.

### Cell Culture and Differentiation

Using previously described methods for adipose tissue digestion in mouse (Sun et al., 2020), we isolated the primary adipose derived stem cells (ADSCs) from embryonic fat tail tissues at E80 by collagenase digestion. Following this, the ADSCs were cultured in DMEM/F12 medium containing 10% FBS and 1% penicillin/streptomycin (growth medium). The differentiation process to generate adipocyte from ADSCs required 6 days in total. Isolated primary cells were cultured in induction medium (growth medium supplemented with 10 ng/ml insulin, 1 μM dexamethasone, 1 μM rosiglitazone and 0.5 mM IBMX) for 2 days, and following this, the induction medium was replaced with keep medium (growth medium supplemented with 10 ng/ml insulin and 1 μM rosiglitazone) for 4 days and the keep medium was refreshed every 2 days. At d6, a large number of droplets were observed by oil red staining. The differentiating cells at d0, d2, d4 and d6 were then used to extract RNA with a standard Trizol method (Invitrogen, Waltham, United States) and examined for expression of *MTFP1* using RT-qPCR.

### Small Interfering RNA Transfection and RT-qPCR Assays

Small interfering RNA (siRNA) transfections were conducted in isolated primary ADSCs. To minimize potential off-targets effect of siRNA, three different siRNAs (siMTFP1-1, siMTFP1-2 and siMTFP1-3) targeting the *MTFP1* gene and a negative control siRNA (siNC) were designed (Supplementary Table S9). Lipofectamine 3,000 (Invitrogen, California, United States) was used with these siRNAs for downregulation of *MTFP1* gene expression according to the manufacturer’s protocol. First, we measured the knockdown efficiency for the three siRNAs and the most significant of these (siMTFP1-1) was used to perform the downstream experiments. The cells culture in growth medium were treated with siMTFP1 to examine its effects on the expression of mitochondrion- and adipogenesis-related genes, and proliferation and migration ability. The RT-qPCR experiment was carried out as previously described (Dong et al., 2020). The sequence of the siRNAs and the primer sequences are provided in Supplementary Table S9. Also, the cells cultured in induction medium and keep medium were treated with siMTFP1 for 6 days to examine its effect on cellular adipogenesis at d6.

### Cell Proliferation, Wound Healing and Oxygen Consumption Rate Assay

Three methods were used to test cell proliferation ability depending on whether *MTFP1* is expressed or knocked down. The first method used a Cell Counting Kit-8 (CCK-8, Sigma-Aldrich, Germany) (Yang et al., 2021). This method involved addition of 10% CCK-8 solution to the cell culture medium, followed by incubation for 1 h at 37°C and colorimetric changes was measured using optical density at 450 nm with a microplate reader (Tecan Infinite 200 Pro, Männedorf, Switzerland). The second method used a different colorimetric assay based on 3-(4,5-dimethylthiazol-2-yl)-2,5-diphenyltetrazolium bromide (MTT, Sigma-Aldrich) (Prabst et al., 2017). MTT solution (10%) was added to the cell culture medium, which was then incubated for 3 h at 37°C. The mixture was discarded, and the same volume of Formazan solution was added for 10 min and the colorimetric change was measured as optical density at 490 nm. The third method involved the use of alamarBlue (Sigma-Aldrich) (Prabst et al., 2017) as a 10% solution added to the cell culture medium, followed by incubation for 2–6 h at 37°C and detection of relative fluorescence units according to the manufacturer’s instructions.

The wound healing assay was performed as follows: the cells were allowed to grow to 100% coverage and a scratch was introduced into the middle of plate; the growth medium was replaced with Opti-MEM (Gibco, Waltham, United States); and photographic images of the scratch at 0 and 12 h were analysed using the ImageJ software (Schneider et al., 2012) for area quantification.

Oxygen consumption rate (OCR) assay was carried out using a BBoxiProbe™ R01 kit (Bestbio, China) to evaluate cell metabolism and mitochondrial state. In brief, this involved the following: 1) cells were cultured in a 96-well black plate with transparent bottom and treated with siMTFP1 for 48 h; 2) refreshing the growth medium and adding 5 μl oxygen fluorescent probe; 3) oxygen blocking buffer was added to prevent external oxygen; 4) relative fluorescence unit (RFU) was detected using a microplate reader every 5 min for 1.5 h.

## Results

### Fat Tail Morphogenesis

**FIGURE 1:**
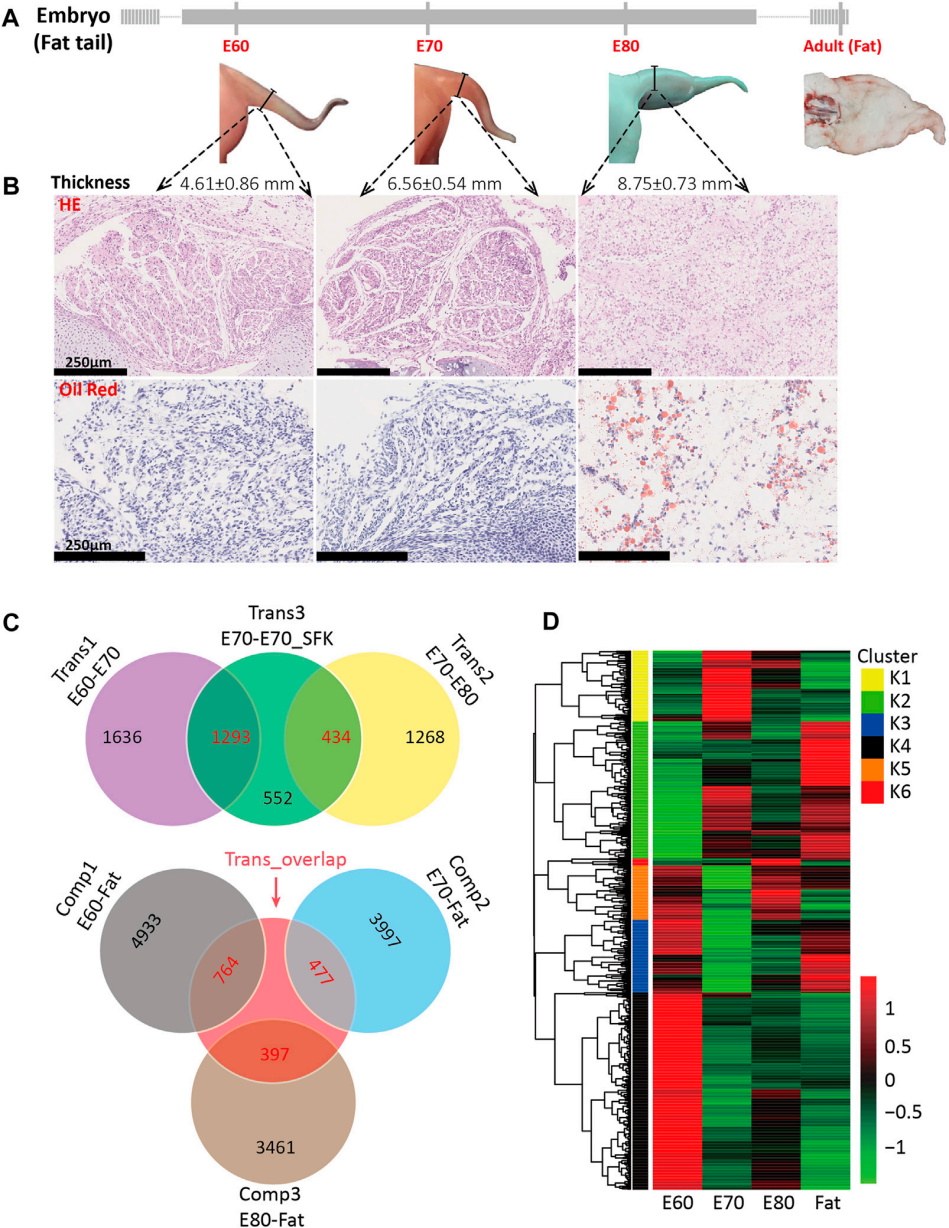
Tail morphogenesis and schematic of DEG comparisons among experimental groups. **(A)** Morphology of tail at four different stages, including E60, E70, E80 and adult, also showing corresponding tail thickness. **(B)** Hematoxylin and eosin (HE) and oil red staining for fat tail tissues at E60, E70, and E80. **(C)** A Venn diagram for the three transition groups and the three comparison groups based on the detected DEGs, including the DEGs between E60 and E70 (trans1), between E70 and E80 (trans2), between E70 and E70_SFK (trans3), between E60 and Fat (comp1), between E70 and Fat (comp2), and between E80 and Fat (comp3). The number of DEGs shared between two groups is shown in red and the number of unique DEGs is shown in black. **(D)** A heatmap for 1,058 candidate genes based on expression at the E60, E70, E80 and Fat stages. These genes overlapped between trans3 and the other five groups individually and can be divided into six clusters based on the Calinsky criterion.

To examine the morphogenesis of the fat-tail trait by external observation, HE and oil red staining were performed for embryonic tail tissue at three time points (E60, E70 and E80). The external morphology of tail tissue at E60 and E70 did not show any change except that the thickness increased gradually with development (Figure 1A). In the sections collected from the E60 and E70 stages, circular preadipocytes accumulated to form regular leaf-like cell masses separated by fibers without fat deposition (Figure 1B and Supplementary Figure S1A). Obvious morphogenesis occurred at E80, including increased thickness, and altered shape characteristics. The sections obtained from E80 showed visible lipids, highly dense adipocytes, and an irregular cell mass shape (Figure 1B and Supplementary Figure S1A). These results demonstrate that morphological changes were caused by fat deposition.

The higher proportion of unilocular adipocytes are mainly distributed in the central area of the cell masses and multilocular adipocytes at the edge (Supplementary Figure S1A), which indicate that the lipid droplets gradually fuse to form a single larger droplet during the morphogenesis of fat tail. In addition, the edge areas of cell masses at E80 were surrounded by fibers developed from veined fiber networks at E60 and E70 (Supplementary Figure S1A). To determine whether specific fat deposition occurred in the tail tissue at E80, we also investigated fat deposition in five additional tissues (muscle, heart, skin, liver and kidney); these results showed that fat droplets stained red were only observed in tail tissue (Supplementary Figure S1B).

### Transcriptome Profiling of Developmental Fat Tail Tissue

To investigate the genomic regulatory network that promote tail development and to obtain a comprehensive view of the transcriptional changes involved in fat-tail morphogenesis, we performed RNA-seq for tail samples from Tan sheep (fat-tail) at four time points, including E60 (*n* = 4), E70 (*n* = 3), E80 (*n* = 4) and adult (Fat) sheep (*n* = 3), as well as Suffolk sheep (thin-tail) at E70 (E70_SFK) (*n* = 4) (Supplementary Figure S2A). Between 32 and 63 million 150-bp paired-end reads were generated for each sample with a mean mapping rate of 78.8% (Supplementary Table S1). After filtering for lowly expressed genes, there were 15,427 genes remaining for subsequent analyses that were expressed in all samples. The expression values of these genes were transformed into FPKM values. The PCA of the expression data partitioned the samples into five broad clusters with the E70_SFK samples dispersed between the E60 and E70 groups (Supplementary Figure S2A). The heatmap generated using all expressed genes showed that E60 and E70_SFK samples clustered on the same branch, with E70 and E80 also sharing a similar transcriptome profile, and with the fat-tail tissue of adult sheep emerging in a single branch (Supplementary Figure S2B).

Subsequently, differentially expressed gene (DEG) analysis among different groups was performed to identify the key regulators involved in tail fat deposition. The comparison between the E60 and E70 stages was termed transition 1 (Trans1), that between E70 and E80 as transition 2 (Trans2), and that between E70 and E70_SFK as transition 3 (Trans3) (Figure 1C). A total of 2,929, 1702 and 1909 DEGs were identified across Trans1, Trans2 and Trans3, respectively, and there were 4,328 unique genes in the union set of DEGs (Supplementary Table S2). There were 1,293 DEGs shared between Trans1 and Trans3 with a totally different expression trend (Figure 1C), including 1,285 DEGs with the opposite direction of expression and eight DEGs with the same direction of expression, an observation consistent with the fact that Trans1 (from E60 to E70) was a process of positive fat tail morphogenesis, while Trans3 (from E70 to E70_SFK) was a comparison between fat and thin-tailed sheep, which represents a negative process of fat tail morphogenesis. There is a significant time gap between the E80 and adult stages; therefore, we examined three different contrasts among the three embryonic time points and the adult time point (Comp1: E60-Fat; Comp2: E70-Fat; and Comp3: E80-Fat). These analyses detected 5,697, 4,474 and 3,858 DEGs for Comp1, Comp2 and Comp3, respectively. The total number of DEGs decreased across the Comp1, Comp2, and Comp3 comparisons, which was reflected in the numbers of DEGs exhibiting increased or decreased expression (Table 1).

**TABLE 1:** DEGs in the three transition groups and the three comparison groups.

DEG expression	Trans1	Trans2	Trans3	Comp1	Comp2	Comp3
Increased	1,120	973	1,063	2,456	2,135	1,804
Decreased	1,809	729	846	3,241	2,339	2,054
Total	2,929	1,702	1,909	5,697	4,474	3,858

Trans1: DEGs, between E60 and E70; Trans2: DEGs, between E70 and E80; Trans3: DEGs, between E70 and E70_SFK; Comp1: DEGs, between E60 and Fat; Comp2: DEGs, between E70 and Fat; Comp3: DEGs, between E80 and Fat.

**FIGURE 2:**
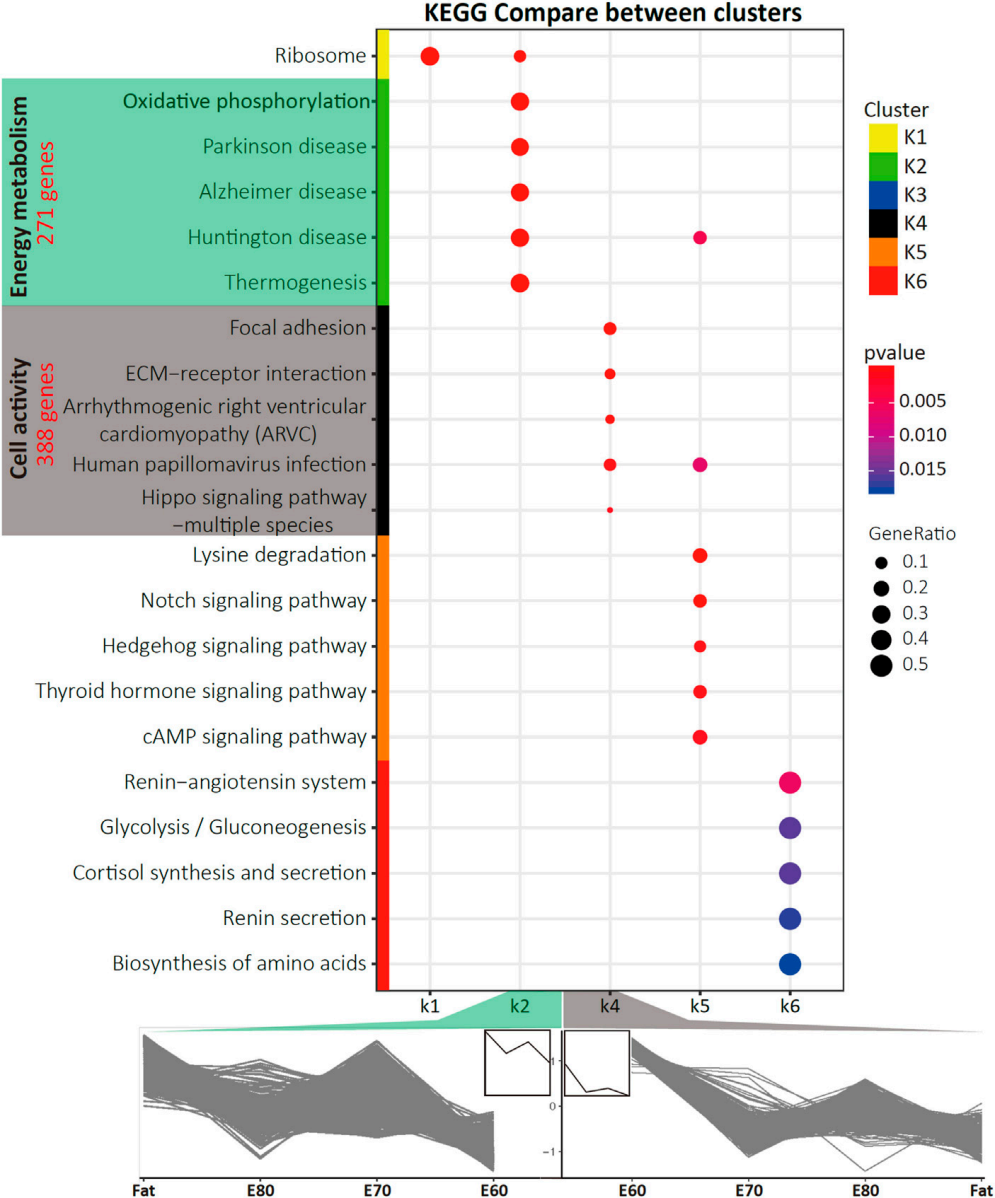
The KEGG pathways that were specifically enriched for each cluster. Also shown is the gene expression time course trends for the K2 and K4 clusters. The pathways that were specifically enriched in K2 and K4 were mainly involved in energy metabolism and cell activity. The number of genes contained in two clusters are indicated in red.

Since Trans3 was the comparison between fat-tail and thin-tail sheep at the same time point, we targeted the Trans3 DEGs that overlapped with the other two transition groups (Trans1 and Trans2) as well as the three comparison groups (Comp1, Comp2, and Comp3) (Figure 1C). A total of 1,058 DEGs were detected (Supplementary Table S2) with different expression patterns across four stages of fat tail development (E60, E70, E80, and Fat), which can be divided into six clusters (K1–K6) by the Calinski-Harabasz Criterion (Calinski and Harabasz, 1974) (Figure 1D, Supplementary Figures S2C, S3). The top two clusters, K2 and K4, containing 271 and 386 genes and accounted for 25.6 and 36.5% of the DEGs, respectively. Cluster K2 represents a cluster of periodic genes that were increased in expression during E60 to E70 and decreased in expression during E70 to E80. Cluster K4, on the other hand, consisted of the periodic genes that exhibited a pattern of expression opposite to cluster K2 (Figure 2).

### Mitochondrial Genes Showing Increased Expression and Enhanced Energy Metabolism Before Fat Differentiation

Through KEGG pathway enrichment analysis for each cluster (Supplementary Figure S3 and Supplementary Table S3), five pathways were specifically enriched in K2, including *Oxidative phosphorylation* and *Thermogenesis* (Figure 2), which are closely related to energy metabolism. The top 10 most significant pathways in K2, in addition to the *Proteasome* and *Ribosome*, shared a subset of DEGs (Supplementary Figure S2D). The pathways overrepresented in K4 are highly associated with cell activity, including *Focal adhesion*, *ECM-receptor interaction*, and *PI3K-Akt signaling pathway* (Figure 2). Furthermore, gene ontology (GO) enrichment analysis was conducted for these two clusters and the top ten most significant terms in each GO category were determined (Supplementary Figure S4 and Supplementary Table S4). Almost all the significant enriched GO terms in K2 were associated with the mitochondrion, such as *Respiratory chain*, *Mitochondrial part*, which was the most significant term, and *Electron transfer activity* (Supplementary Figure S4).

**FIGURE 3:**
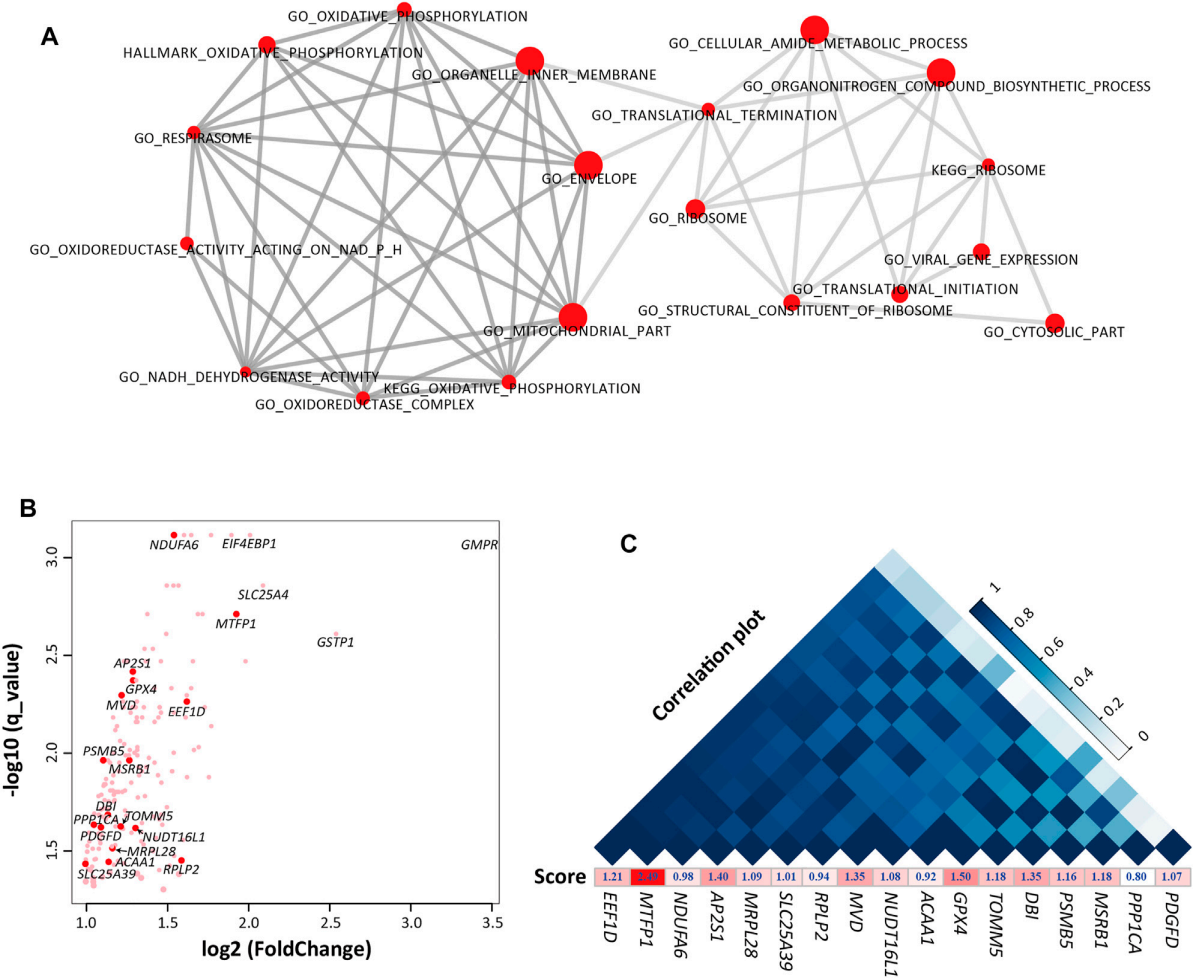
The gene sets and genes that were enriched in E70 compared with E70_SFK by Gene Set Enrichment Analysis (GSEA). **(A)** The top 10 most significant gene sets in each functional group. Left, energy metabolism-related gene sets. Right, regulation of transcription-related gene sets. **(B)** Expression fold-changes between E70 and E70_SFK and the corresponding q values for the genes enriched in the gene sets that were upregulated at E70 (*n* = 183). The red data points indicate the 17 genes that have been previously reported. **(C)** An expression correlation plot (top) for the 17 previously reported genes according to their expression for E60, E70, E80, Fat and E70_SFK and ranked by their correlation coefficient. The enrichment score for each gene obtained using GSEA is also displayed below.

In order to further understand the function of these genes, we performed gene set enrichment analysis (GSEA) (Subramanian et al., 2005) using the gene expression data between E70 and E70_SFK. GSEA is an enrichment method that evaluates transcriptome profile data at the level of gene sets. As a result, 37 gene sets were specifically enriched at E70 (*q* < 0.05), which can be mainly divided into two groups according to their biological function (Supplementary Table S5). One group related to different components of the mitochondrion as well as energy metabolism, and another group is involved in ribosome function and translation. The top 10 most significant gene sets in each part are displayed in Figure 3A. However, the 52 gene sets enriched at E70_SFK were difficult to assign to a primary functional category, and the GO term *DNA binding transcription factor activity* exhibited the highest NES score (Supplementary Figure S5B).

Furthermore, 183 genes have been identified in the 37 gene sets that were enriched at E70 by leading edge gene analysis compared with E70_SFK (Subramanian et al., 2005) (Figure 3B and Supplementary Figure S5C). Sixty-nine DEGs were associated with various components of the mitochondrion (Supplementary Table S7), including *TIMM50*, *MPC1*, *MPC2*, and *TOMM5* genes; other genes were involved in membrane transport in mitochondrion and subunits of NADH dehydrogenase (*NDUFA*, *NDUFB*, *NDUFC*, and *NDUFS*). There are also some genes encoding proteins with a functional role in regulating gene expression and cellular activities. The *GNG11* and *GNG5* genes encode proteins belonging to the G protein gamma family that are involved in various transmembrane signaling systems (Gautam et al., 1989). The *POLR2G*, *POLR2I*, and *POLR3K* genes encode subunits of the DNA-dependent RNA polymerase that synthesizes mRNA precursors and many functional non-coding RNAs (Ujvari and Luse, 2006). The *NME2* and *NME4* genes (Hu et al., 2019) encode proteins within the NDK (nucleoside diphosphate kinase) family and have multiple functions in cellular energetics, signaling, proliferation, differentiation, and tumor invasion (Boissan et al., 2009). In addition, there were four genes that were most overexpressed in E70 compared with E70_SFK, including *GMPR*, *GSTP1*, *SLC25A4*, *EIF4EBP* genes, which may encode important regulators of nucleotide metabolism (Figure 3B).

Among these 183 enriched genes, 17 genes have been reported in previous tail phenotype related studies in sheep (Wang et al., 2014; Li et al., 2018; Ma et al., 2018; Bakhtiarizadeh et al., 2019; Kalds et al., 2021; Wang et al., 2021; Zhang et al., 2021). Expression of these genes fluctuated from the E60 to Fat stages and was significantly higher at E70 than E70_SFK (Supplementary Figures S6A,S6B). The reported genes can be divided into two groups according to the pathways they were assigned to (Supplementary Table S8): energy metabolism related genes (*MTFP1*, *ACAA1*, *GPX4*, *MRPL28*, *MSRB1*, *NDUFA6*, *PPP1CA*, *SLC25A39*, and *TOMM5*) and regulation of transcription related genes (*AP2S1*, *DBI*, *EEF1D*, *MVD*, *NUDT16L1*, *PDGFD*, *PSMB5* and *RPLP2*). The expression of *MTFP1* and *EEF1D* genes was highly positively correlated to other previously reported genes (Figure 3C). It has been reported that mitochondrial fission process protein 1 (encoded by *MTFP1*) is one of the key regulators of mitochondria fission, and involved in cell apoptosis, carcinogenesis, and tumor progression. In particular, the expression of *MTFP1* was most significantly different between E70 and E70_SFK with the highest enrichment score (Figures 3B,C); this warrants further research for its functional role in adipogenesis *in vitro*.

### Knockdown of *MTFP1* Promote Adipogenesis

**FIGURE 4:**
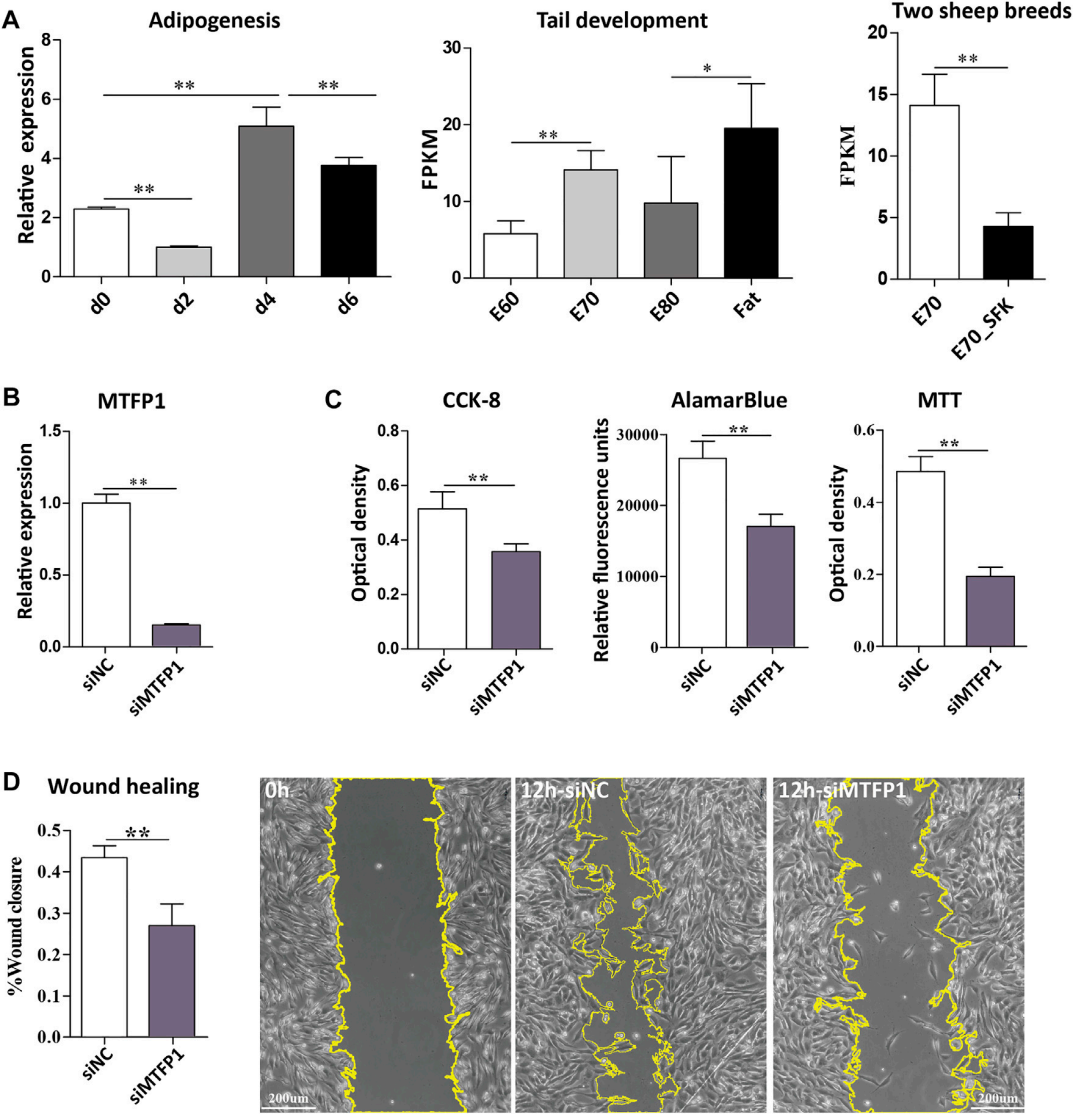
Expression of the *MTFP1* gene and the effect of siRNA-induced knockdown of *MTFP1* on cell proliferation and migration. **(A)** Relative expression of *MTFP1* at d0, d2, d4 and d6 of adipocyte differentiation and absolute expression of *MTFP1* in tail tissues at different developmental stages. **(B)** Silencing efficiency of *siMTFP1*. **(C)** The influence of *siMTFP1* on cell proliferation detected using CCK-8, MTT, and alamarBlue methods. **(D)** The effect of siRNA-induced *MTFP1* knockdown on wound healing as measured by wound closure rate. The micrographs with yellow highlighting represent the wound area of cells treated with siMTFP1 and siNC at 0 and 12 h. Error bars represent mean ± SD. *p < 0.05, **p < 0.01.

Firstly, we found that the expression of *MTFP1* fluctuated during the process of ADSCs differentiation (d0, d2, d4 and d6) and the expression at the late stage is higher than that at the early stages, which is consistent with the expression trend in fat-tail tissues from the E60 to Fat stages (Figure 4A). Small interfering RNA (siRNA) was used to downregulate the expression *MTFP1* and to investigate its role in proliferation, migration and adipogenesis. Three siRNAs were designed to specifically downregulate expression of *MTFP1* and the siRNA with the most effective knockdown ability (siMTFP1-1) was selected to perform downstream assays and minimize potential off-target effects (Supplementary Figure S6C). After treatment with *siMTFP1*, the gene expression decreased to 20% of normal expression (Figure 4B). Three methods, including CCK-8, alamarBlue and MTT, jointly showed that knockdown of *MTFP1* inhibited cell proliferation (Figure 4C), which was in concordance with the results of the wound healing experiment showing that downregulation of this gene significantly reduced cell migration ability (Figure 4D). Furthermore, we examined the expression of other mitochondrion-related genes that also has been identified in this study (Supplementary Table S7) and adipogenesis-related genes.

**FIGURE 5:**
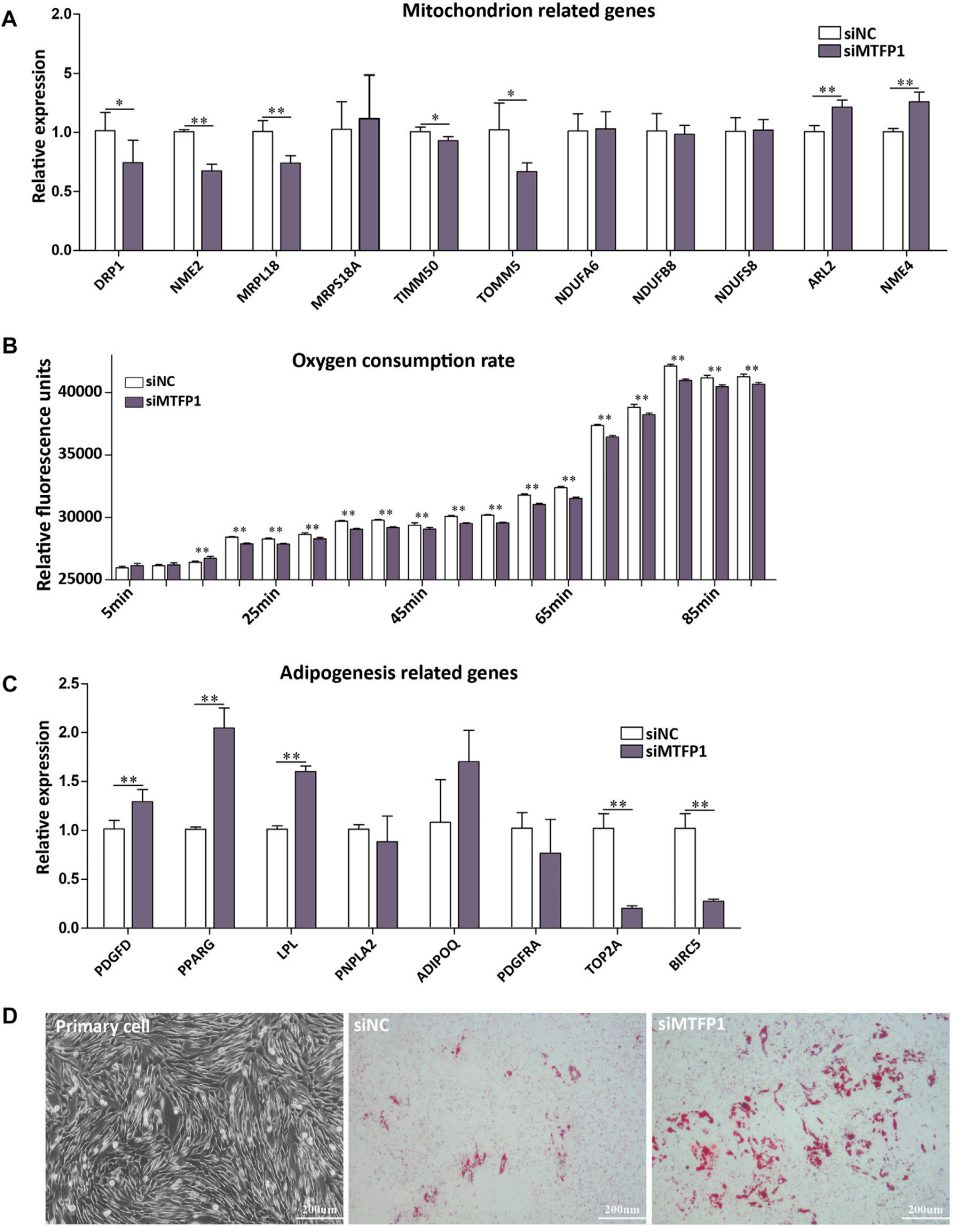
**(A)** The effect of siRNA-induced *MTFP1* knockdown on the expression of genes associated with mitochondrial function, including *DRP1*, *NME2*, *MRPL18*, *MRPS18A*, *TIMM50*, *TOMM5*, *NDUFA6*, *NDUFB8*, *NDUFS8*, *ARL2* and *NME4*. **(B)** The effect of siRNA-induced *MTFP1* knockdown on oxygen consumption within cell. The effect of siRNA-induced *MTFP1* knockdown on the expression of genes associated with adipogenesis **(C)**, including: *PDGFD*, *PPARG*, *LPL*, *PNPLA2*, *ADIPOQ*, *PDGFRA*, *TOP2A* and *BIRC2*, as well as its effect on adipocyte differentiation *in vitro* **(D)**. Error bars represent mean ± SD. *p < 0.05, **p < 0.01.

Knockdown of *MTFP1* resulted in decreased expression of *DRP1*, *NME2*, *MRPL18*, *TIMM50*, and *TOMM5*, which are genes involved in the mitochondrial membrane, ribosome and membrane protein (Smirnov et al., 2011; Morita et al., 2017; Chaudhuri et al., 2021). The genes (*NDUFA6*, *NDUFB8,* and *NDUFS8*) that encode subunits of NADH dehydrogenase (Stroud et al., 2016) retained their original expression levels. Besides, expression of *ARL2* and *NME4* was upregulated (Figure 5A). Although it has been reported that expression of *NME4* can stimulate respiratory ATP regeneration (Tokarska-Schlattner et al., 2008), the oxygen consumption ability within these cells was still inhibited after silencing *MTFP1*, which may caused by decreased cell number (Figure 5B). For adipogenesis-related genes, decreased expression of *MTFP1* induced upregulation of adipocyte-related genes (*PPARG* and *LPL*) and downregulation of progenitor-associated genes (*TOP2A* and *BIRC5*), reflecting a positive effect on adipogenesis after silencing *MTFP1* (Figure 5C) We also treated the ADSCs with *siMTFP1* during cell differentiation, which significantly promoted fat deposition (Figure 5D).

## Discussion

In this study, we showed clearly that the key stage of fat tail development is E70, before which genes and pathways involving in metabolism pathways were significantly increased in activity, resulting in fat tail morphogenesis from E70 to E80. Furthermore, 183 genes have been identified that may play important roles in fat tail development and 17 of these genes have been reported in previous studies. Knockdown of *MTFP1*, a key gene in mitochondrion organization, was shown to inhibit cell proliferation and migration ability, as well as promote adipogenesis *in vitro*.

Based on morphological observation and histological examination, the morphology and microstructure of embryonic tail tissue changed significantly from E70 to E80. Preadipocytes were observed to be accumulated into regular cell masses at E70, while visible lipid droplets and high-density adipocytes were evident at E80 (Figure 1B). However, the most significant transcriptional difference existed between the E60 and E70 stages, such that this comparison generated the largest number of DEGs (Figure 1C). The biological differences between E60 and E70 were also evident in the PCA and clustering analysis (Supplementary Figures S2A,S2B). Furthermore, the KEGG pathways and GO terms enriched in the K2 cluster, which consisted of genes upregulated at E70, were related to energy metabolism, including *Oxidative phosphorylation*, *Thermogenesis*, and *Components of the mitochondrion* (Figure 2 and Supplementary Figure S4). Compared with E70_SFK, the thin tail samples, mitochondrial gene sets were also specifically enriched at E70 by GSEA analysis (Figure 3). In particular, genes exhibiting increased expression at E70 encoded subunits of NADH dehydrogenase, and proteins involved with the respiration, fission and fusion processes of mitochondria, which therefore reflected mitochondria proliferation, ATP production and increased metabolism.

Our results showed that increased expression of mitochondrion-associated genes from E60 to E70 would lead to proliferation of mitochondria and promote oxidative phosphorylation to generate energy and synthesis of cellular components for cell differentiation and fat deposition. After the E70 stage, preadipocytes differentiated into adipocytes with excessive fat deposition, which result in morphological changes of tail tissue, comparable to what was observed at E80 (Figure 1A). Therefore, we deduced that the 70-day stage of gestation is the key time point for fat-tail development with transcriptional changes leading to fat deposition and morphogenesis of fat-tail tissues.

Importantly, 17 genes detected in this study were identified in previous studies (Figure 3C, Supplementary Table S7). For example, *PDGFD* and *PPP1CA* are two genes that were identified in some population genetics studies (Zhu et al., 2016; Dong et al., 2020; Li et al., 2020). In our study, *PDGFD*, *MSRB1* as well as the other 17 DEGs were enriched in the *Response to oxidative stress* pathway, which is involved in reactive oxygen species (ROS) generation and therefore in a wide range of cellular processes (Martindale and Holbrook, 2002). The *EEF1D* gene together with *EEF1B2*, *EIF3I* and *EIF4EBP1* encode important components of the eukaryotic translation elongation and initiation factor complex and are essential for several steps in the initiation of protein synthesis (McLachlan et al., 2019). Using human cells, the MTFP1 protein has been shown to be essential for maintenance of mitochondrial integrity; perturbation of *MTFP1* expression leads to morphological changes in mitochondria and influences cell survival (Tondera et al., 2004, 2005; Wang et al., 2017).

The process of mitochondria fission is mainly affected by the products of the *DRP1*, *MTFP1* and *FIS1* genes (Tondera et al., 2005), and it has been shown that MTFP1 serves as an essential regulator of mitochondrial fission through the modulation of DRP1 phosphorylation and recruitment to the mitochondrion (Morita et al., 2017). However, there are no reports concerning the function of *MTFP1* in adipogenesis. Here, we conducted a preliminary exploration of MTFP1 function in fat tail development through siRNA-mediated knockdown of the *MTFP1* gene (Figures 4, 5). Knockdown of *MTFP1* significantly repressed mitochondria generation, cell proliferation and migration, which has been observed previously (Tondera et al., 2005,^2004^), and oxygen consumption within cells. Meanwhile, downregulation of *MTFP1* would be expected to promote adipogenesis.

We confirmed that fat tail tissue is a type of white adipose tissue because *UCP1* gene expression, a canonical brown adipocyte marker that localizes at the mitochondrial inner membrane, was not observed at all stages of development examined, including E60, E70, E80 and adult (Chouchani et al., 2019). The expression pattern of adipocyte markers or adipogenesis regulators can be used to monitor cellular adipogenesis. In this regard, the expression of adipocyte markers (including *CEBPA*, *PPARG*, *CIDEA*, *FABP4*, *LPL* and *ADIPOQ*) (Mildmay-White and Khan, 2017) increased significantly from E60 and E80, especially at the adult stage (Supplementary Figure S7A), where high expression levels were observed for these genes. At the same time, mesenchymal stem cell/adipocyte progenitor markers (including *CDCA8*, *CCNB1*, *PDGFRA*, *TOP2A* and *BIRC2*) (Merrick et al., 2019) exhibited an opposite expression trend, decreasing significantly from E60 to the adult stage (Supplementary Figure S7B).

There are two biological effects of *MTFP1* knockdown: inhibition of mitochondrial activity (Figure 5A); and induction of expression of adipocyte markers (*LPL* and *PPARG*) with concomitant decrease in the expression of MSC markers (*TOP2A* and *BIRC5*). Both of these mechanisms are important. Adipogenesis is a process involving crosstalk between preadipocyte proliferation and differentiation, such that good proliferative ability is required for terminal differentiation of ADSCs (Tang and Lane, 2012). There are two published studies showing that overexpression of miR-199a-5p in porcine preadipocytes and miR-200b in 3T3-L1 cell line promotes cell proliferation, which impairs adipogenic ability (Shen et al., 2018; Shi et al., 2014), thereby indicating opposing roles for certain genes in cell proliferation and differentiation.

As a member of the peroxisome proliferator-activated receptor (PPAR) family, PPARG is an essential regulator of adipogenesis, playing a crucial role in cellular differentiation, lipid accumulation, insulin sensitivity, and triglyceride metabolism (Barak et al., 1999). Absence of *PPARG* gene expression within cells lead to dysregulation of adipogenic conversion (Rosen, 2005). In addition, it has been shown that ectopic expression of *PPARG* can influence mitochondrial biogenesis, and the inflammation response across the urothelial barrier in bladder epithelial cells (Liu et al., 2019). Lipoprotein lipase (LPL) is the key enzyme in triglyceride metabolism, involved in fatty acid synthesis, lipid transport and conversion between different types of lipid (Roberts et al., 2022). Increased expression of *LPL* and *PPARG* can therefore directly regulate and modulate fat synthesis and deposition within cell.

In summary, we hypothesized that, initially, at d0 of differentiating ADSCs or at E70 of fat-tail tissue (Figure 4A), high expression of *MTFP1* maintains preadipocyte proliferation and cellular energy production. Following this, attenuated expression of *MTFP1* enhances initialization of the cell differentiation process, as we observed decreasing expression from d0 to d2 *in vitro* and from E70 to E80 *in vivo*. Finally, in successfully differentiated adipocytes, *MTFP1* expression returns to a relatively high level to maintain energy production and cellular synthesis processes necessary for fat deposition.

## Conclusion

We ascertained the microstructure and morphology of fat tail in sheep before and after fat deposition. The key time point for fat tail development was E70, before which genes and pathways related to energy metabolism were significantly upregulated to facilitate energy production for cell differentiation, resulting in tail fat deposition at E80, earlier than other tissues. There are 17 DEGs that were specifically upregulated at E70 and that have also been reported in previous studies. Knockdown of the most significant gene (*MTFP1*) can repress cell proliferation, migration and oxygen consumption, while promoting the process of adipogenesis *in vitro*.

## Data Availability

The datasets presented in this study can be found in online repositories. The names of the repository/repositories and accession number(s) can be found in the article/Supplementary Material.

## Supplementary Material

SUPPLEMENTARY FIGURE 1
**(A)** Microstructure of tail tissue at E60 (top) and E80 (bottom). Black ellipses correspond to magnified areas on the right; black arrows shown unilocular adipocytes; and red arrow indicate the boundary of the cell mass at E80. **(B)** Oil red staining of six tissues (tail, muscle, heart, skin, liver, and kidney) at E80.

SUPPLEMENTARY FIGURE 2
**(A)** The PCA diagram for all samples used for RNA-Seq based on FPKM value of all expressed genes. **(B)** The heatmap diagram for all samples used for RNA-seq based on FPKM values for all expressed genes, with samples assigned into three branches. Genes were removed that exhibited mean FPKM values < 0.5 in at least one group. **(C)** The Calinski criteria demonstrates that the optimum number of clusters for candidate DEGs is six (K1-6). **(D)** The top 10 most significant KEGG pathways in the K2 cluster share common genes.

SUPPLEMENTARY FIGURE 3 The gene expression time course trends across E60, E70, E80 to Fat and the top 10 most significant pathways in each cluster (K1-6).

SUPPLEMENTARY FIGURE 4 The bar plot for the top ten most significantly enriched GO terms within the biological process, molecular function, and cell component categories for the K2 (top) and K4 (bottom) clusters.

SUPPLEMENTARY FIGURE 5
**(A)** GSEA plots for the most significant gene sets upregulated at E70 in three molecular signature databases (GO, KEGG and Hallmark). **(B)** GSEA plot for the most significant gene set upregulated at E70_SFK. **(C)** The enrichment score and FPKM value of genes (*n* = 183) that were enriched in gene sets that were upregulated in the fat tail tissue at E70 among significantly enriched gene sets and samples, respectively.

SUPPLEMENTARY FIGURE 6 The expression values for 17 previously reported genes from E60, E70, E80 to Fat, and between E70 and E70_SFK and the interfering efficiency of three siRNAs targeting *MTFP1*. **(A)** Genes related to energy metabolism (*n* = 9), including *MTFP1*, *GPX4*, *TOMM5*, *MSRB1*, *MRPL28*, *SLC25A39*, *NDUFA6*, *ACAA1* and *PPP1CA*. **(B)** Genes related to regulation of transcription (*n* = 8), including *AP2S1*, *MVD*, *DBI*, *EEF1D*, *PSMB5*, *NUDT16L1*, *PDGFD* and *RPLP2*. **(C)** The interfering efficiency of three siRNAs (siMTFP1-1 to 3) targeting *MTFP1*, and the siMTFP1-1 exhibited the most efficient effect was selected to perform downstream assays. Error bars represent mean ± SD. **p* < 0.05, ***p* < 0.01.

SUPPLEMENTARY FIGURE 7 The expression values for adipogenic genes from E60, E70, E80 to Fat, and between E70 and E70_SFK. **(A)** adipocytes markers (*n* = 6), including *CIDEA*, *FABP4*, *CEBPA*, *PPARG*, *LPL* and *ADIPOQ*. **(B)** Mesenchymal stem cell (MSC) markers and preadipocyte markers (*n* = 5), including *CDCA8*, *CCNB1*, *PDGFRA*, *TOP2A* and *BIRC2*.
