# Supplementary figures and images for: Transcriptome Profiling of Developing Ovine Fat Tail Tissue Reveals an Important Role for MTFP1 in Regulation of Adipogenesis
Source: Front Cell Dev Biol. 2022 Mar 8;10:839731. doi: 10.3389/fcell.2022.839731 (PMC8957931; doi:10.3389/fcell.2022.839731)

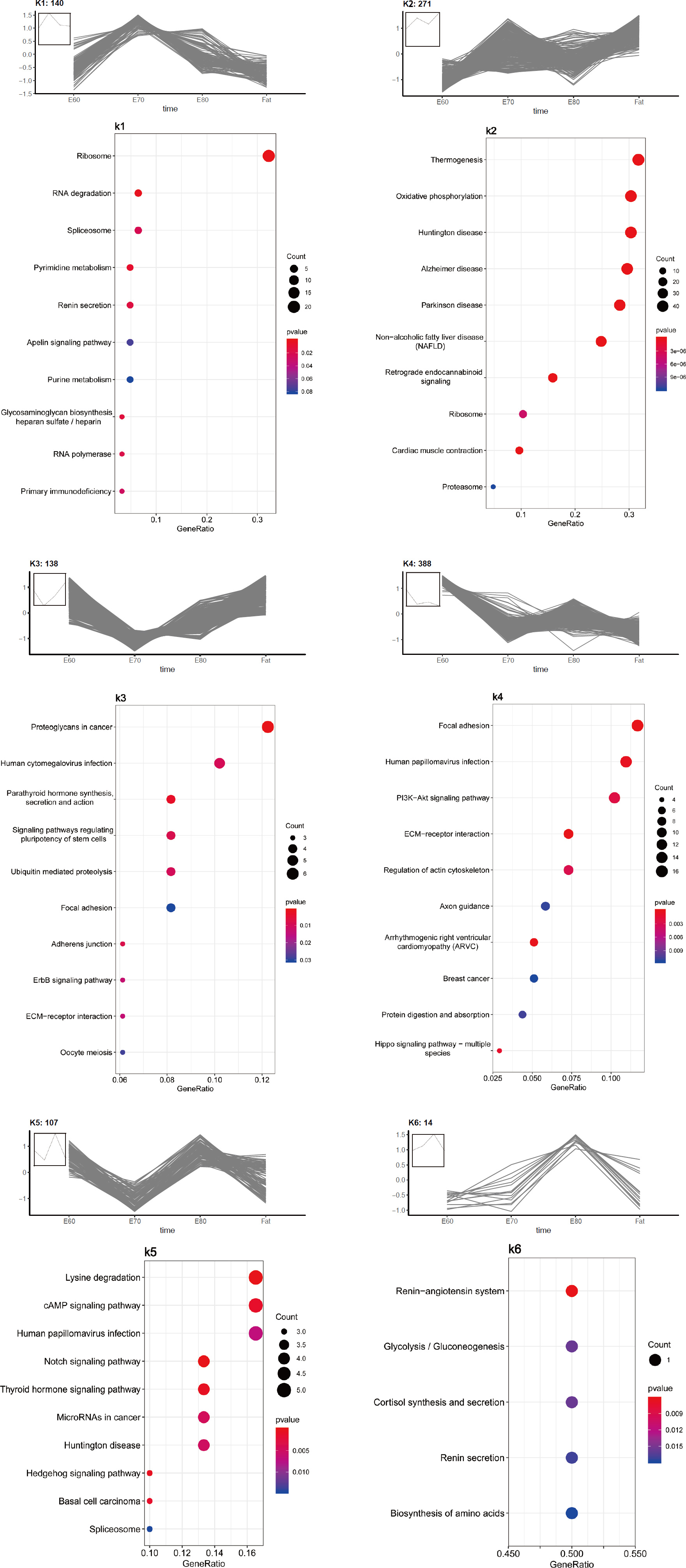

Supplement: Supplementary file 1 [file Image3.JPEG]

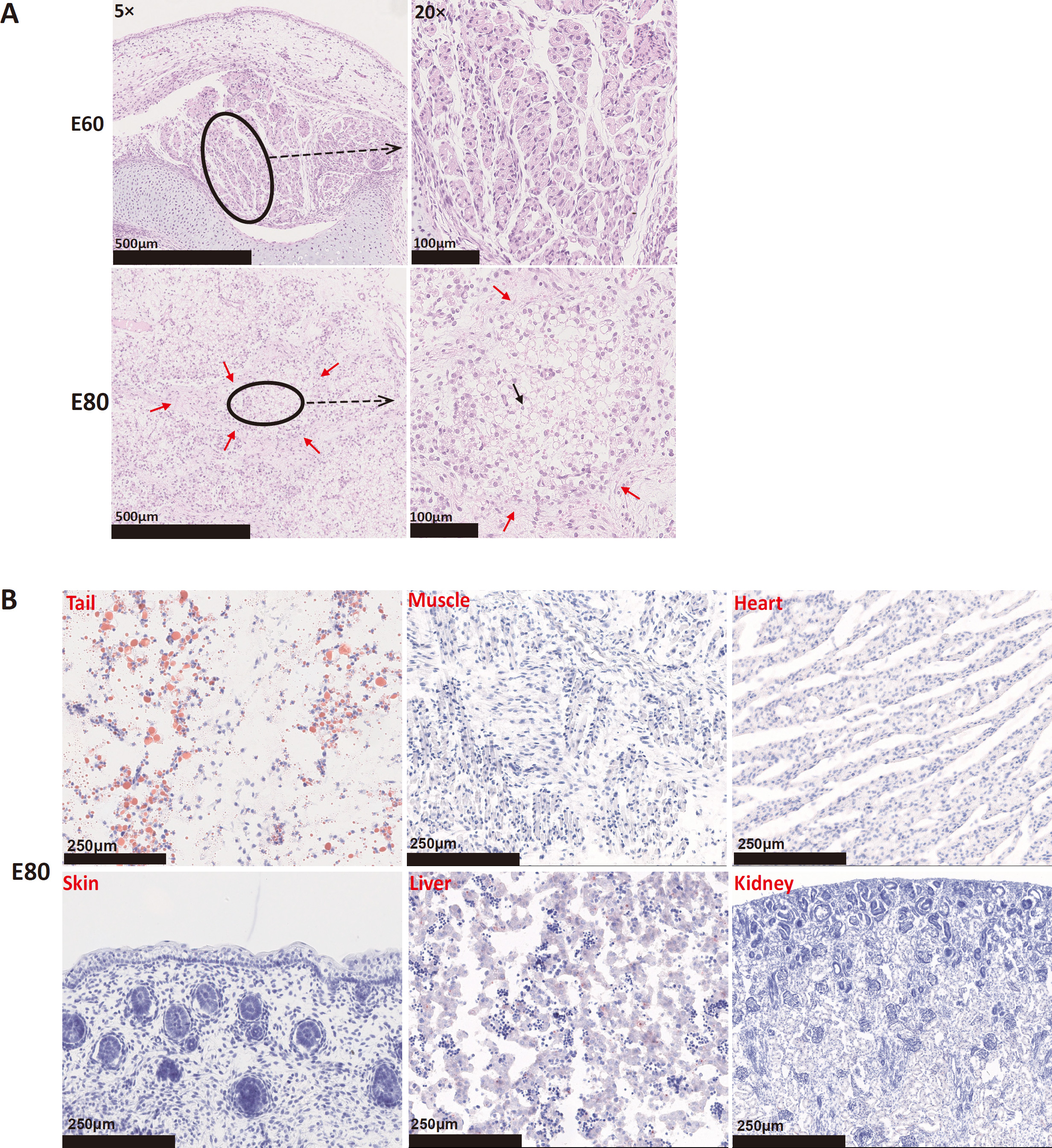

Supplement: Supplementary file 2 [file Image1.JPEG]

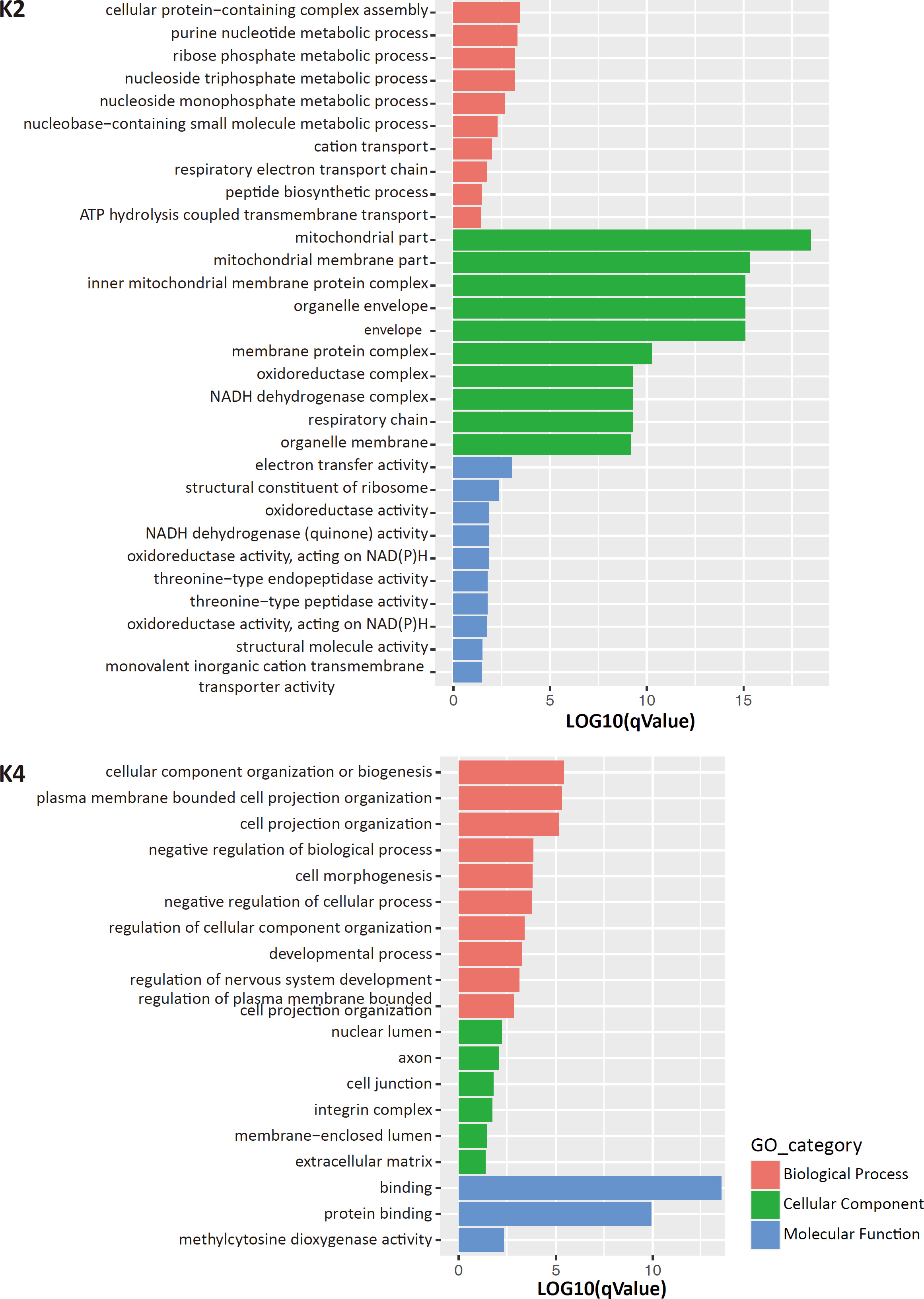

Supplement: Supplementary file 3 [file Image4.JPEG]

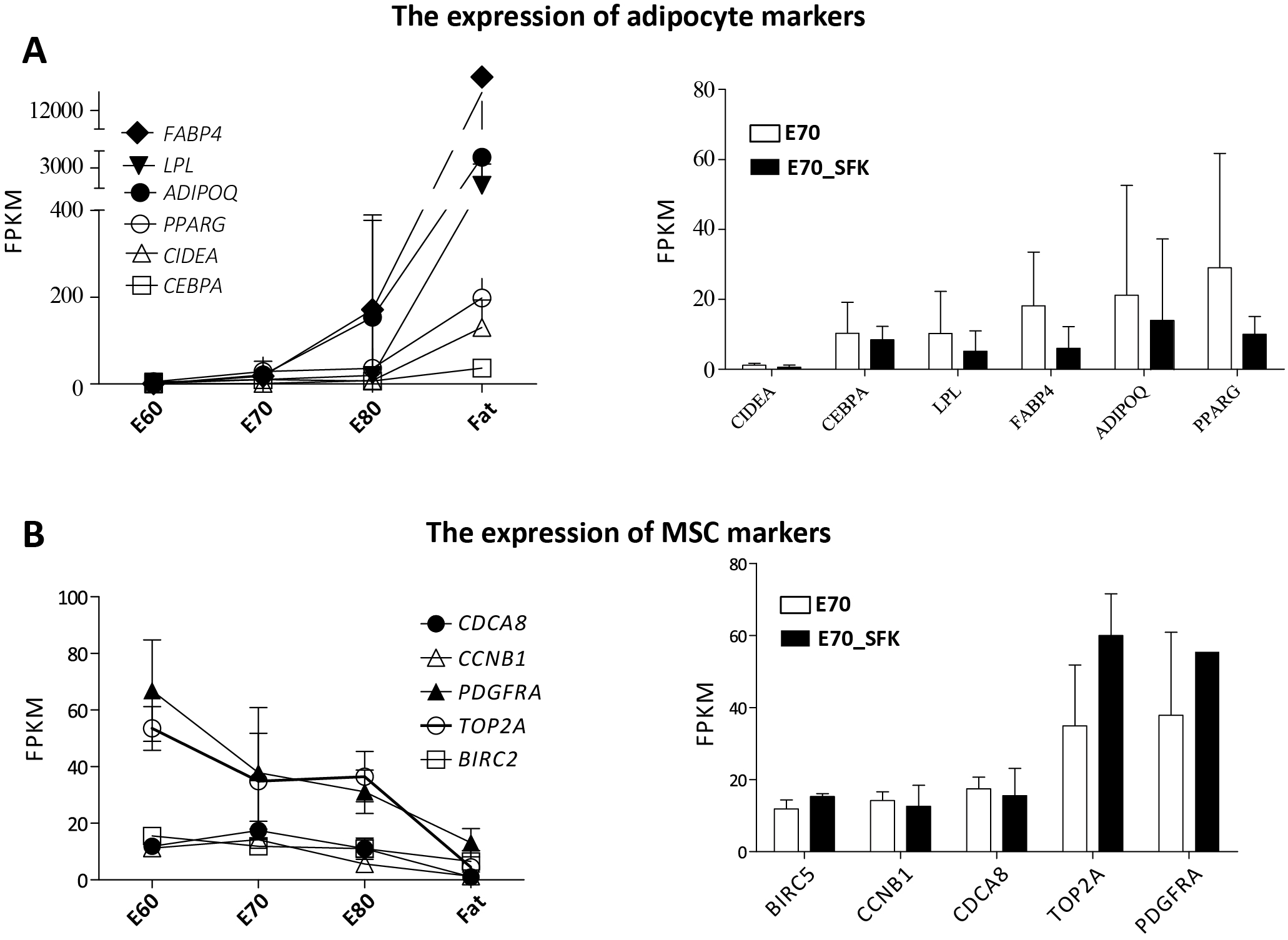

Supplement: Supplementary file 4 [file Image7.JPEG]

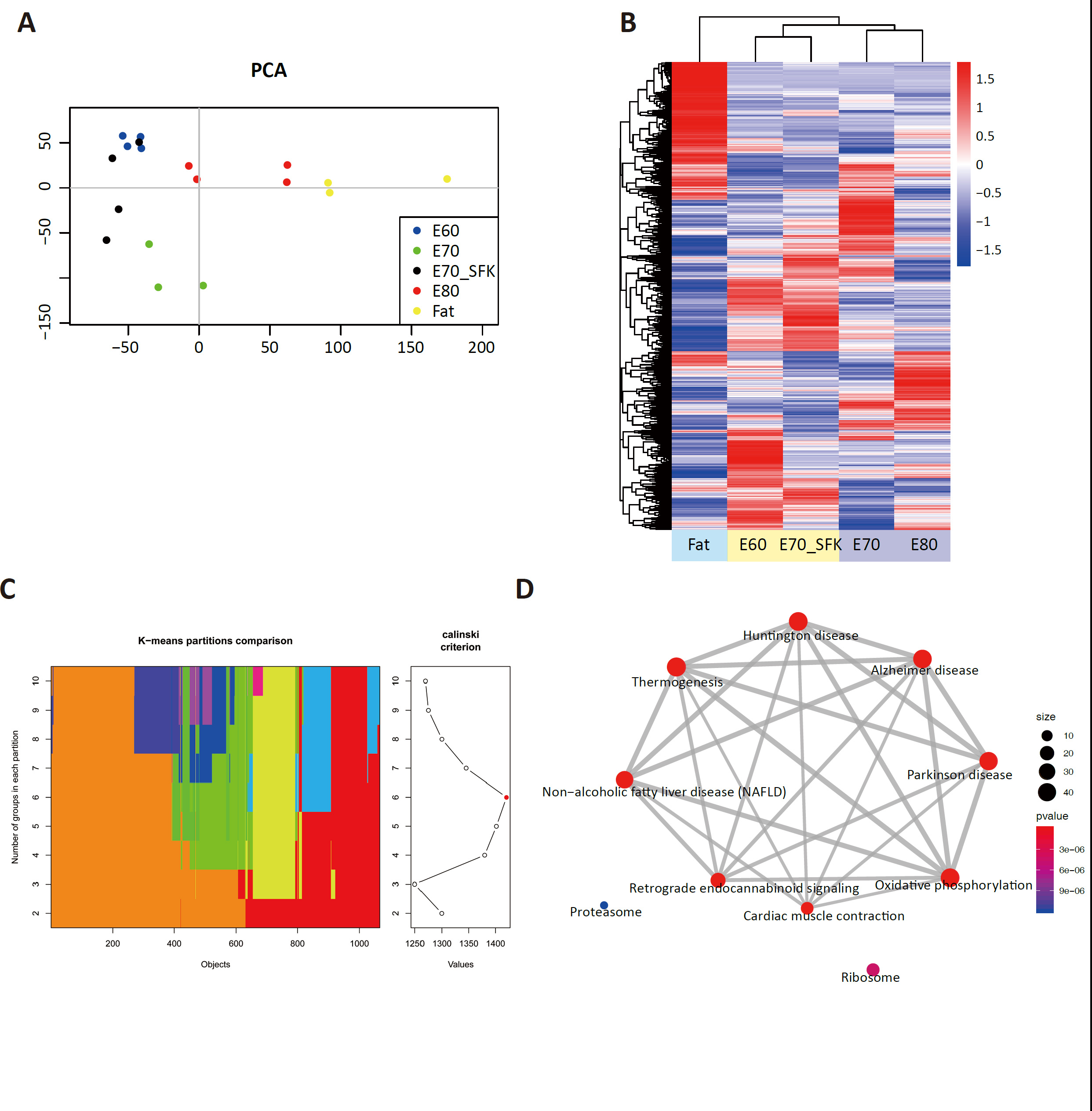

Supplement: Supplementary file 5 [file Image2.JPEG]

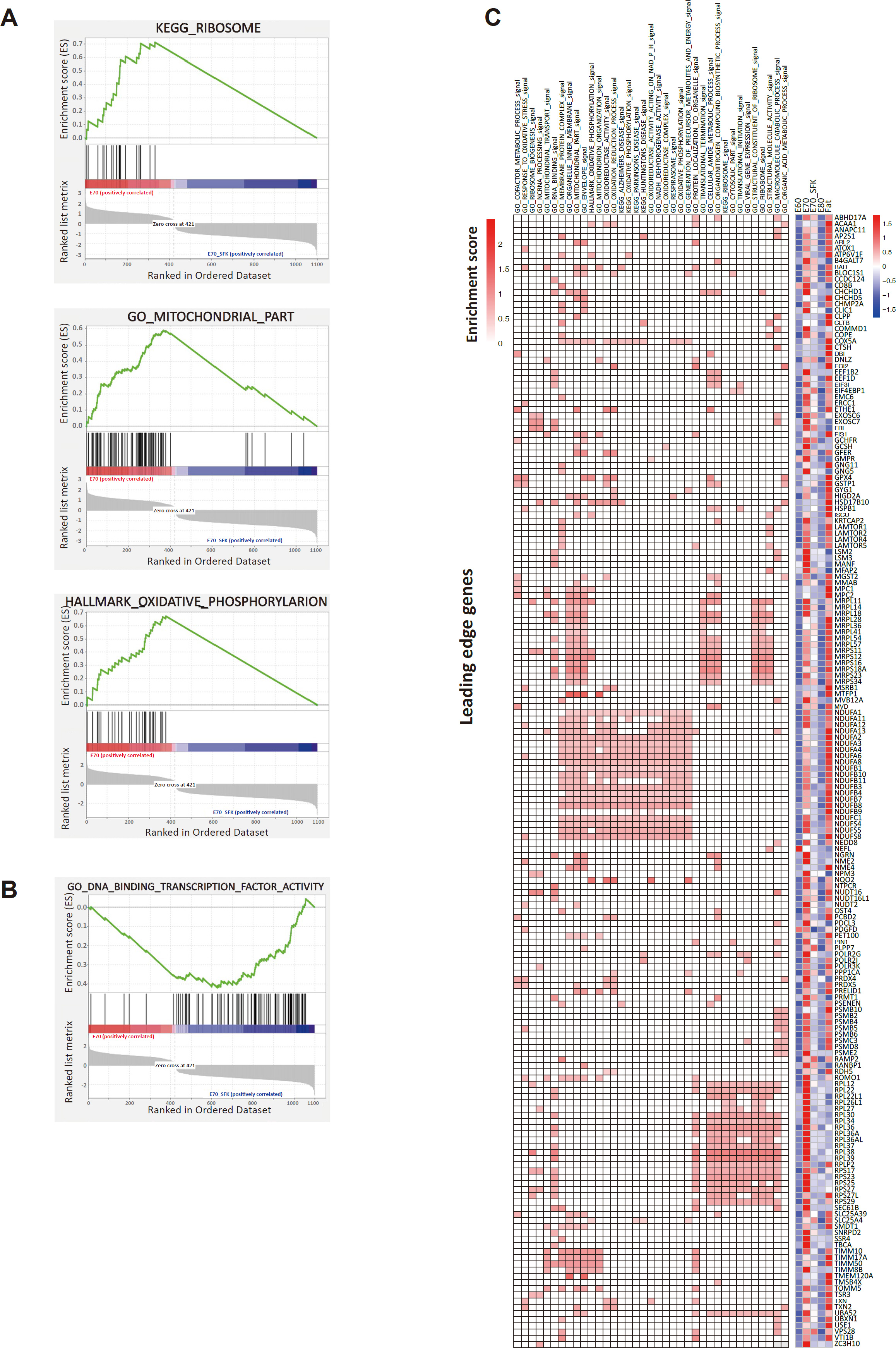

Supplement: Supplementary file 6 [file Image5.JPEG]

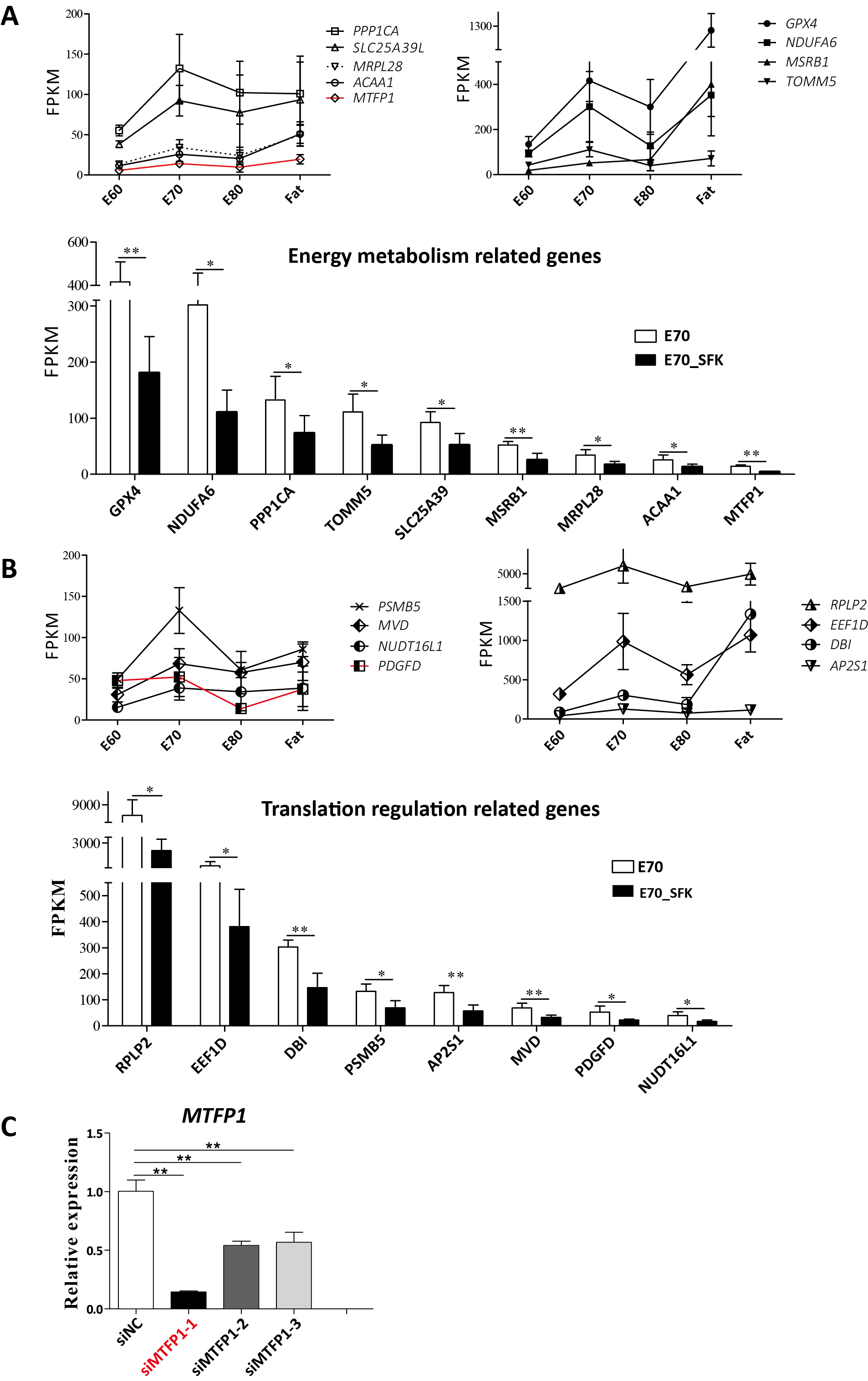

Supplement: Supplementary file 7 [file Image6.JPEG]
